# Donor-Control of Scavenging Food Webs at the Land-Ocean Interface

**DOI:** 10.1371/journal.pone.0068221

**Published:** 2013-06-27

**Authors:** Thomas A. Schlacher, Simone Strydom, Rod M. Connolly, David Schoeman

**Affiliations:** 1 Faculty of Science, University of the Sunshine Coast, Maroochydore, Queensland, Australia; 2 Australian Rivers Institute – Coast & Estuaries, Griffith University, Gold Coast, Southport, Queensland, Australia; University of Southampton, United Kingdom

## Abstract

Food webs near the interface of adjacent ecosystems are potentially subsidised by the flux of organic matter across system boundaries. Such subsidies, including carrion of marine provenance, are predicted to be instrumental on open-coast sandy shores where in situ productivity is low and boundaries are long and highly permeable to imports from the sea. We tested the effect of carrion supply on the structure of consumer dynamics in a beach-dune system using broad-scale, repeated additions of carcasses at the strandline of an exposed beach in eastern Australia. Carrion inputs increased the abundance of large invertebrate scavengers (ghost crabs, *Ocypode* spp.), a numerical response most strongly expressed by the largest size-class in the population, and likely due to aggregative behaviour in the short term. Consumption of carrion at the beach-dune interface was rapid and efficient, driven overwhelmingly by facultative avian scavengers. This guild of vertebrate scavengers comprises several species of birds of prey (sea eagles, kites), crows and gulls, which reacted strongly to concentrations of fish carrion, creating hotspots of intense scavenging activity along the shoreline. Detection of carrion effects at several trophic levels suggests that feeding links arising from carcasses shape the architecture and dynamics of food webs at the land-ocean interface.

## Introduction

Fluxes of materials, energy, nutrients and organisms are a fundamental feature of many ecological boundaries [1]. These exchanges are widespread, creating inputs of allochthonous matter that can constitute subsidies for food webs in the recipient systems [2,3]. Such subsidies are disproportionally important in ecosystems that have low *in situ* productivity, such as sandy beaches, arctic regions, and deserts [4–6].

Organic matter is imported into subsidised food webs as either plant detritus or as animal carcasses. Animal carcasses (i.e. carrion) are an abundant and widespread resource: many ecosystems contain large numbers of dead animals that have died from non-predation events [7,8]. These rich carrion resources are exploited by a diverse and highly evolved guild of scavengers [9], giving rise to a large, but often underappreciated, scavenging pathways in food webs [10].

Systems with large perimeter-to-area ratios (e.g. streams, riparian forests, small islands, beaches) respond more strongly to trophic subsidies due to their greater propensity to receive inputs across their long boundaries [11]. Similarly, environments that are relatively open to neighbouring ecosystems (i.e. have permeable boundaries) often show strong responses to subsidies [3]. Both of these boundary conditions apply to sandy beaches of open-coasts where nutrients and matter are readily exchanged across the open and extended boundaries between the sea and land [12]. Arguably, sandy beaches of open coasts are archetypal interface regions: they form one of the biosphere’s longest ecotones where the oceans abut the land along 70% of the globe’s ice-free coastline [13]. In terms of ecosystem energetics, beach foods webs are underpinned by marine imports and hence illustrate trophic subsidies *par excellence*, including the processing of marine carrion [14,15].

Material transfer across ecosystem boundaries, including the beach-dune interface, requires a vector to move matter – either biological or physical. On beaches, physical vectors that move marine matter onshore are primarily wind, currents, tides and waves [16]. These physical forces can deposit large amounts of stranded marine matter on the shore (i.e. wrack, carrion, flotsam and jetsam) that forms a critical structural component of beach habitats and crucial resource for beach consumers [17,18].

Theoretical expectations therefore are that food webs of open-coast sandy beaches contain scavengers that have evolved to be efficient consumers of stranded carrion resources. We aimed to test three aspects of this ecological context: i) trajectories of change in abundance of scavengers following altered availability of food resources, ii) capacity for carrion processing in the ecosystem, and iii) carrion as donor control of top-level consumers.

## Methods

### Study site

**Figure 1:**
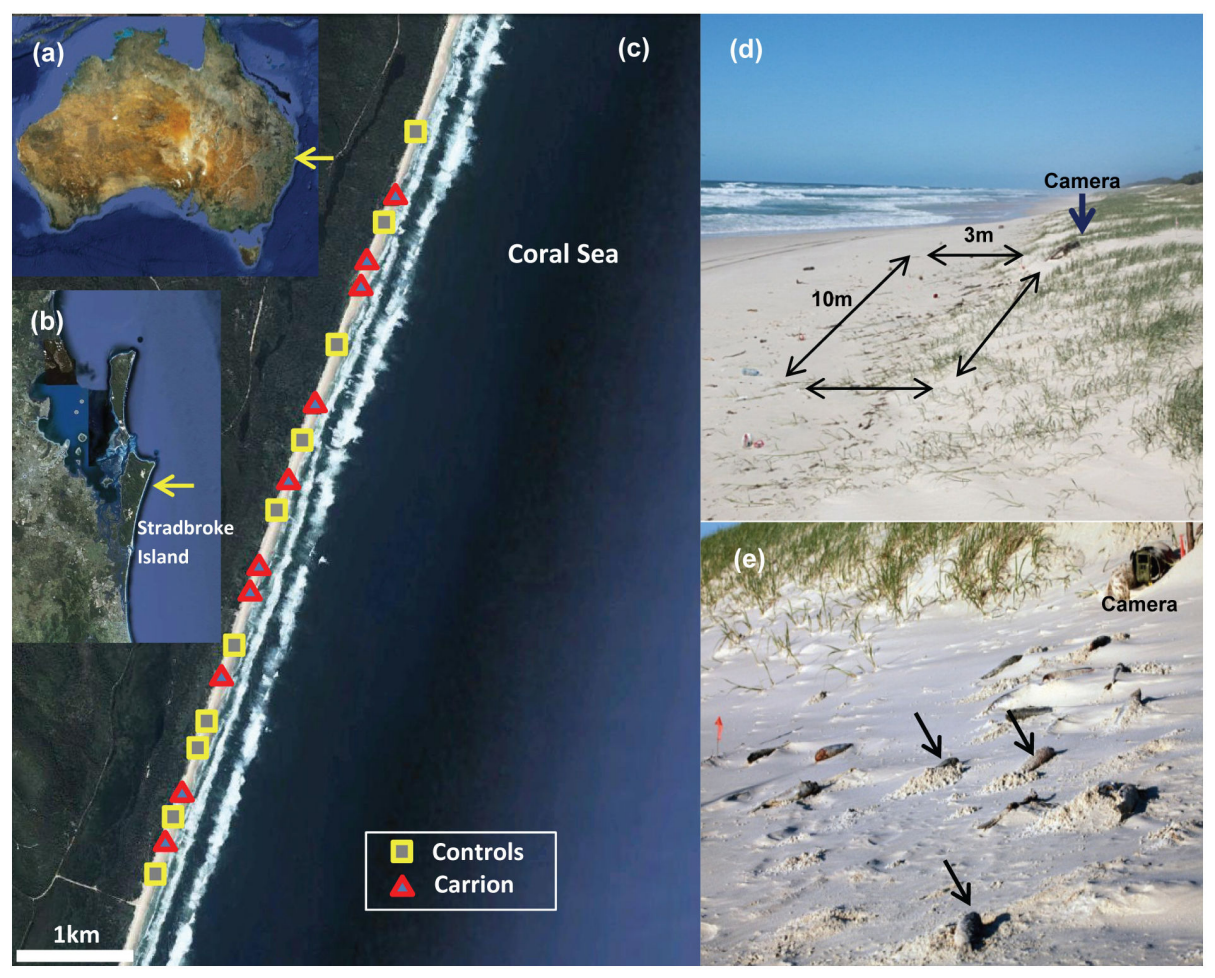
Location of the study area in eastern Australia (a) on the ocean-exposed shore of North Stradbroke Island (b), where experimental plots were established along a 5 km stretch of beach (c). Experimental plots (3 × 10 m) were located at the base of the dunes (d). Fish (arrows in panel e) were added to treatment plots. Motion and IR-triggered cameras were placed in the southern top corner of plots to record larger vertebrate scavengers.

The response of scavengers to carrion subsidies was tested experimentally on an exposed stretch of sandy beach on North Stradbroke Island, Australia (Figure 1). The site is representative of the open-coast beaches along much of the east coast of Australia, being of the intermediate morphodynamic type, with modal wave heights of 1 to 2 m, a 50 to 80 m wide beach-face, and medium-grained sands [19–22]. The beach was selected because it is not backed by urban development and hence interference of experiments by dogs and beach visitors was likely to be lower than elsewhere in the region.

### Experimental design

Carrion availability was experimentally manipulated along a 5 km long stretch of beach, containing 20 experimental plots (10 treatment and 10 control plots). Plots were dispersed along the beach, with distances between plots randomised to fall within a range of 100 to 500 m (actual mean distance between plots: 255 m, se = 29 m, min = 107 m, max = 485 m). Treatments were randomly allocated to plots, with the constraint that no more than two consecutive plots of the same type were acceptable (Figure 1d). Plots measured 3 m (across-shore) x 10 m (along-shore), adopting the same dimensions as used in several papers on ghost crabs in the region [23–27], with the upper edge of the plots aligned with the base of the foredunes (Figure 1).

The experiment comprised two phases: i) ‘BEFORE’, consisting of measurements of scavenger density over 4 weeks (01 to 30 March 2012) without any manipulation of carrion levels in either treatment or control plots, followed by ii) ‘CARRION PULSE’, representing one week (31 March to 7 April 2012) of experimental manipulation of fish carcass numbers in treatment plots. Densities of scavengers were measured on 7 days before the experimental augmentation of carrion (i.e. ‘*before*’ phase) and on 8 days after carrion had been added to treatment plots (i.e. ‘carrion pulse’ phase).

Carrion levels were manipulated repeatedly by adding whole fish carcasses (flathead mullet, *Mugil* *cephalus* ) to treatment plots. Mullet was chosen for the experiment because, as carrion, they form part of the diet of ghost crabs and raptors, the primary invertebrate and vertebrate scavengers in the experiments [28–30]. The fish were caught in the surf zone of local beaches by a commercial fisher. They were, on average, 36 cm long (mean TL = 36.4 cm, se = 0.3, n = 30) and weighed about 500 g (mean wet weight = 499 g, se = 9.4, n = 30).

On 8 days when carrion was added to treatment plots, ten fish were scattered haphazardly across each treatment plot. About 5 kg of fish carrion was added to each plot per day, and the total amount of fish augmented over the course of the experiment was 359 kg (Table 1).

**Table 1:** Summary of experimental setup, scavenger responses, and carrion turnover.

**Design**	
Length of experimental area	5 km of ocean-exposed beach
No. of experimental plots	20 (10 treatment, 10 controls)
Dispersion of plots	107 to 485 m (randomised between adjacent plots)
Number of days when scavengers were counted in the field	n = 15 (7 before and 8 during the experimental addition of carrion)
**Invertebrate scavenger response**	
No. of burrow openings counted and measured	14,783
Change in ghost crab density following carrion additions	overall: 1.7 times (up to 3 times in individual plots)
Largest density increase	16 times for largest crabs (> 50 mm)
**Carrion turnover**	
No. of fish carcasses used in experiment	720
Biomass of fish carrion added to beach	359 kg
No. of fish carcasses scavenged	698 (97%)
Carrion biomass consumed	348 kg (8.7 kg day^-1^ km^-1^)
No. days with complete removal of all fish	5

The fish were added two hours before sunset. The next morning, within two hours of sunrise, the following variables were recorded for each plot: i) number and diameter of ghost crab burrows (see below), ii) number of fish carcasses remaining, and iii) presence of vertebrate scavengers (foxes, birds) or their tracks.

### Scavengers

The primary ecological response to carrion augmentation measured in the experiment was change in the density of ghost crabs (*Ocypode spp.*). Ghost crabs were chosen as the target scavenger species because they are the most abundant invertebrate scavengers on sandy beaches in the region, and are facultative scavengers with a catholic diet [31]. Ghost crab density was quantified by measuring burrow openings on the beach surface; this technique has become a standard method for abundance estimates of ghost crabs on sandy shores [32–35].

Two species of ghost crabs, *Ocypodeceratophthalmus* and *Ocypodecordimanus* , occur on the beaches of Stradbroke Island, but their burrow openings cannot be distinguished with confidence. Burrow counts are therefore reported at the genus level following Schlacher and Lucrezi [27]. On some days strong winds were likely to have obscured burrow openings, and counts from those days (n = 3) were excluded from the analysis.

During pilot studies, five species of avian scavengers were observed to consume fish carrion on the beach. There were three raptors (white bellied sea eagle ( *Haliaeetus* *leucogaster* ), whistling kite ( *Haliastur* *sphenurus* ) and brahminy kite ( *Haliastur* *indus* )), as well as torresian crows ( *Corvus* *orru* ) and silver gulls ( *Chroicocephalus* *novaehollandiae* ). Although each of these species is known to consume carrion [36–39], their response to changing carrion resource availability on beaches has not been documented to date. Other vertebrate scavengers which could potentially react to augmented carcass stocks (also based on our pilot observations) are red foxes ( *Vulpes* *vulpes* , Canidae) and lace monitors ( *Varanus* *varius* , Varanidae).

We recorded vertebrate scavengers with the now-standardised technique of automated cameras [4,40,41]. Digital cameras with IR and motion detectors (ScoutGuard 8.0MP) were placed at plots during the carrion pulse phase of the experiment. Camera deployment was not possible during the weeks leading up to the carrion additions due to high risk of theft and vandalism. Cameras were placed near the upper edge of the experimental plots to capture as much as possible of the plot in the camera’s field of view (Figure 1e). Local flotsam and jetsam items were used to camouflage cameras.

### Data analysis

The analytical design follows ideas proposed by Underwood [42], described by Keough and Quinn [43] as Multiple Before-After, Control-Impact (MBACI). Here, we have fixed effects for ‘PHASE’ (before vs. carrion pulse) and ‘TREATMENT’ (carrion addition vs. control plots) as well as an interaction term for PHASE × TREATMENT. Keough and Quinn [43] nested TIME (an ordered factor indicating the sequence of sampling) in PHASE, and PLOT (the individual experimental plots visited at each TIME) within TREATMENT. They then proceeded with conventional ANOVA. We preferred to treat these latter two factors explicitly as random effects, however, because we intend to generalize results across times before and during carrion addition, and across plots in control and treatment sites. For this reason, we used the (generalized) linear mixed model ((G) LMM) framework provided by the lme4 package [44] in the statistical software R [45]. Besides explicitly incorporating both fixed and random effects, (G) LMMS also accommodate missing observations (resulting from plots washed out during the course of the experiment), allow non-Gaussian data structures, such as those associated with counts, and facilitate implicit nesting of random effects within the model structure. In all cases, we fitted models and assessed diagnostics according to the standard methods described by Pinheiro and Bates [46], Bolker et al. [47], and Zuur [48]. Significance of factors and coefficients were assessed using a combination of likelihood-ratio and Wald tests.

When considering mean burrow diameter of ghost crabs per plot as the response variable, we fitted a standard LMM, and estimated asymptotic p-values with which to test hypotheses regarding fixed-effects coefficients. Because this approach tends to underestimate p-values, these were checked using Markov Chain Monte Carlo (MCMC) sampling to compute 95% highest posterior density intervals [49]. For burrow counts, we assumed a Poisson error structure and fitted a GLMM, including a per-observation random effect to account for over-dispersion. In this instance, we relied on Wald z-tests output by the analysis for hypothesis tests regarding fixed-effects coefficients.

For the vertebrate scavengers, we extracted three complementary metrics of scavenging behaviour from the time-stamped, camera records: i) time to detection (i.e. time elapsed between experimental carcass placement and observed scavenging activity), ii) duration of scavenging bouts (i.e. time spent at the carcass during each distinct scavenging event), and iii) time of scavenging events over a 24h period (i.e. the temporal distribution of scavenging activity). Mean values of detection time and duration were compared among species with ANOVA, followed by Tukey’s HSD post-hoc test when significant main effects were present [50,51]. Uniformity of the temporal distribution of feeding activity, distributed on a circular scale, was assessed with Watson’s U^2^ test [52].

## Results

### Carrion turnover

Carrion removal from the beach was near complete: of the 720 fish carcasses experimentally added to treatment plots over 8 days, 698, or 97%, were completely consumed (Table 1). Scavengers consumed 348 kg of the 359 kg of fish added: this translates to a carrion consumption rate of 8.7 kg of fish biomass per day per km of shoreline. Complete removal of all carrion (i.e. no fish carcasses remaining in treatment plots) was recorded on 5 of the 8 days when we monitored carrion turnover. No carrion remained in 4 plots at the end of the experiment (Table 1).

### Invertebrate scavengers

**Figure 2:**
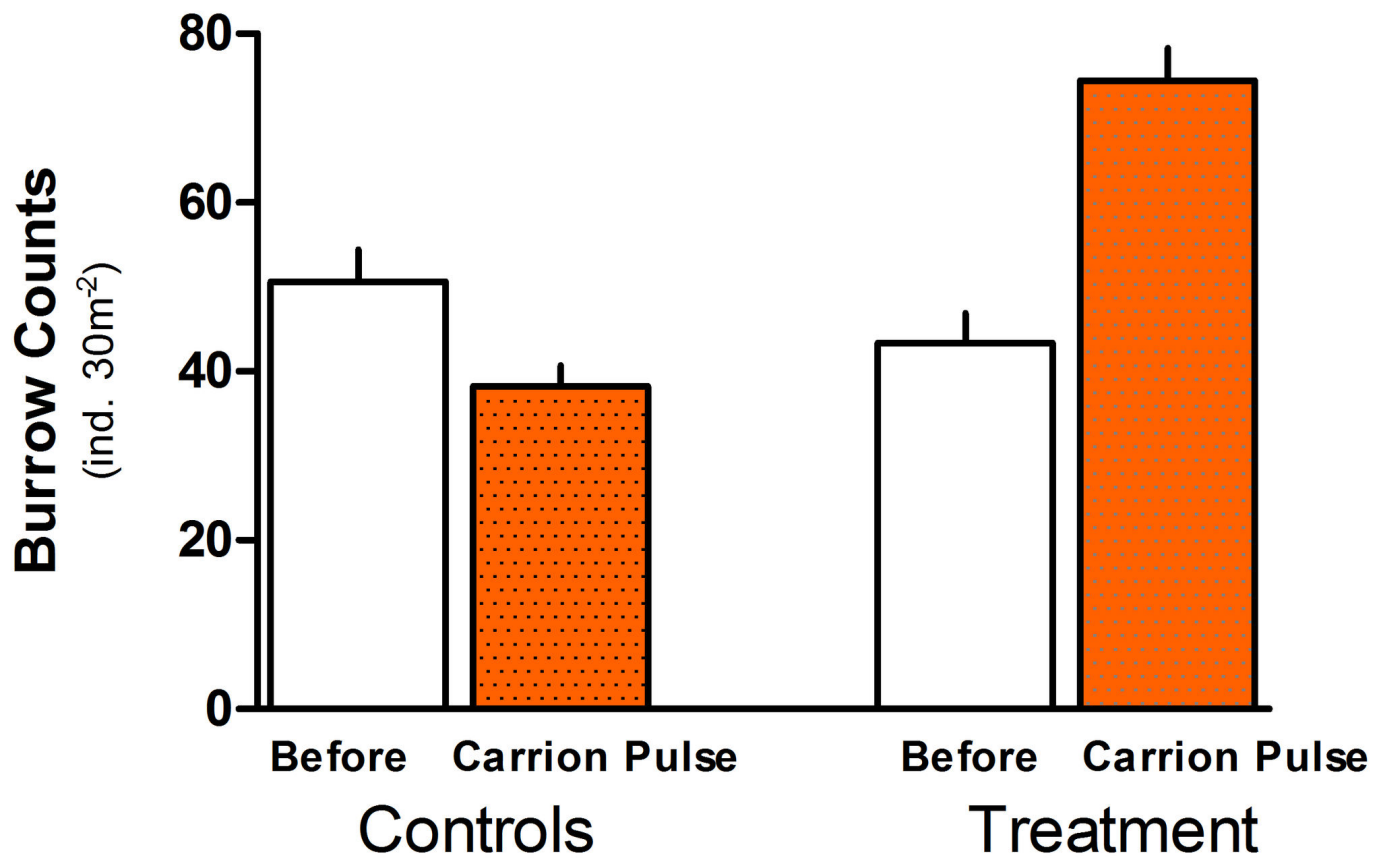
Comparison of the density (mean, SE) of ghost crabs before (open bars) and during (filled bars) the experimental augmentation of carrion availability. Fish carcasses were added to treatment plots only during the ‘carrion pulse’ phase of the experiment.

**Figure 3:**
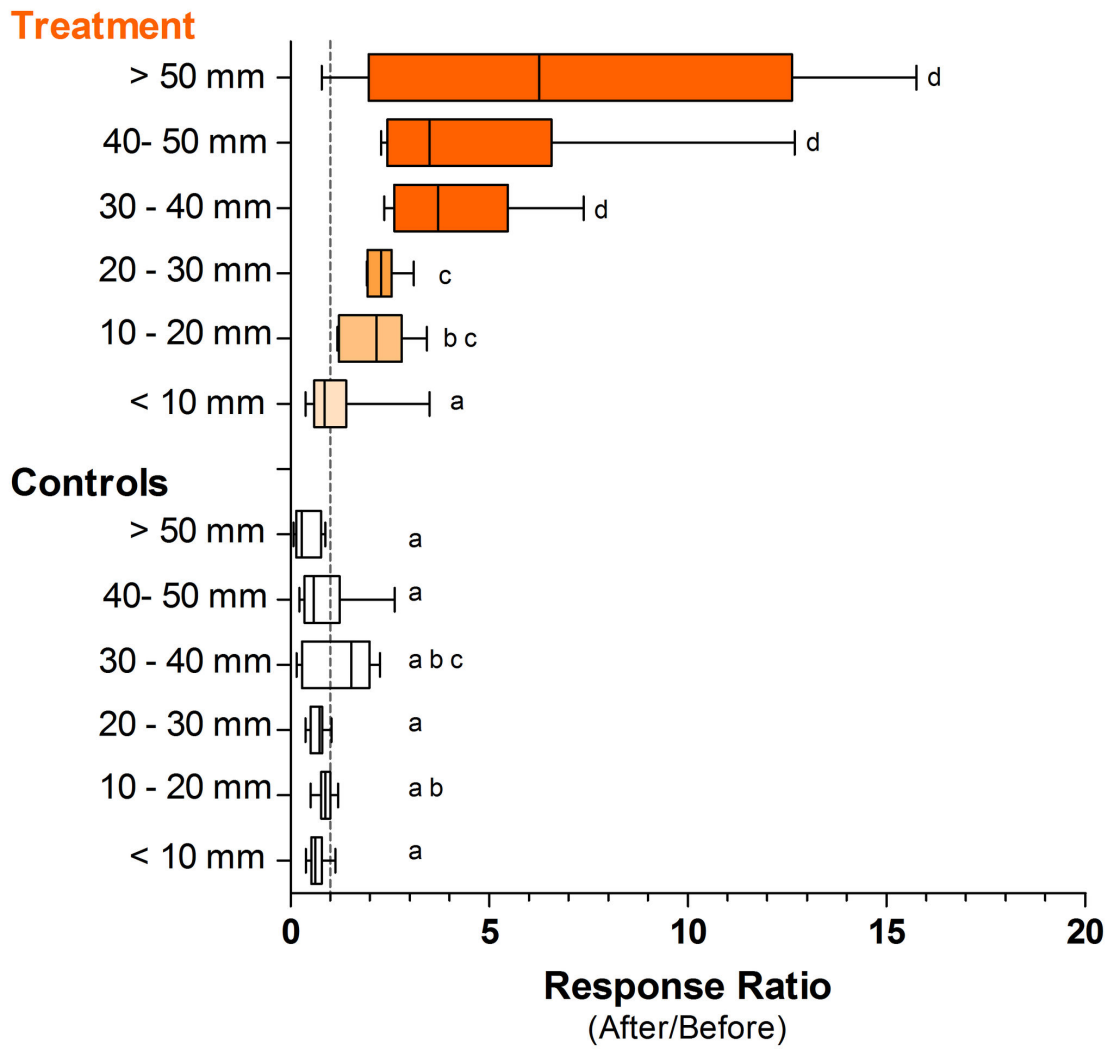
Density response to experimental augmentation of fish carcasses by different size classes of ghost crabs. Measure of organism size is the burrow opening diameter of ghost crabs. The metric of density change is the ratio of mean densities per plot before and after the addition of carrion (i.e., response ratio = mean ind. m^-2^ plot_i_ size-class_x_ BEFORE) / mean ind. m^-2^ plot_i_ size-class_x_ AFTER). Letters denote homogeneous groups as defined by SNK post-hoc testing following significant main effects in ANOVA. Boxes encompass the interquartile range (Q1 to Q3) and whiskers are the 10^th^ and 90^th^ percentile.

For both burrow density and diameter, the full (generalized) mixed model proved most parsimonious, retaining all random and fixed effects (Table 2). In both instances, the PHASE x TREATMENT interaction was highly significant (p < 0.0001), indicating that ghost crabs responded strongly to the addition of carrion (Figure 2, Table 2). At the treatment plots, both burrow density (p = 0.005) and diameter (p = 0.021) increased significantly during carrion addition. Specifically, ghost crabs were, on average, about twice as abundant in treatment plots during the experimental addition of carrion (mean burrow counts: before = 43 ± 3.5 ind. 30 m^-2^; during carrion pulse = 74 ± 3.8 ind. 30 m^-2^), and these burrow diameters were on average 2.3 mm larger (Table 3, Figure 3, MCMC 95% highest posterior density interval: 0.82-3.85 mm). By contrast, at the control sites, neither burrow density (p = 0.281) nor diameter (asymptotic p = 0.299) changed significantly over the corresponding period. Thus, larger numbers of ghost crab burrows between phases of the experiment recorded in the treatment plots were not a general environmental effect unrelated to carrion availability.

**Table 2:** Summary of Wald χ^2^ tests for fixed effects in the final (full) (generalized) mixed-effects model of ghost-crab responses to carrion additions.

	**Burrow Diameter**				**Burrow Density**		
	Chi-sq	df	p		Chi-sq	df	p
Phase	0.4284	1	0.5128		0.8598	1	0.3538
Treatment	20.7509	1	< 0.0001		9.1636	1	0.0025
Phase x Treatment	59.9673	1	< 0.0001		66.1660	1	< 0.0001

**Table 3:** Summary statistics of burrow opening diameters of ghost crabs in relation to phases of the experiment (before and during the addition of fish carrion) in treatment and control plots.

	**n**	**mean**	**se**	**Q25**	**median**	**Q75**
**Control**						
Before	3496	14.17	0.13	9	12	18
Carrion Pulse	2829	13.68	0.13	10	11	17
**Treatment**						
Before	2949	14.29	0.14	9	12	18
Carrion Pulse	5509	17.06	0.14	10	14	20

### Vertebrate scavengers

**Figure 4:**
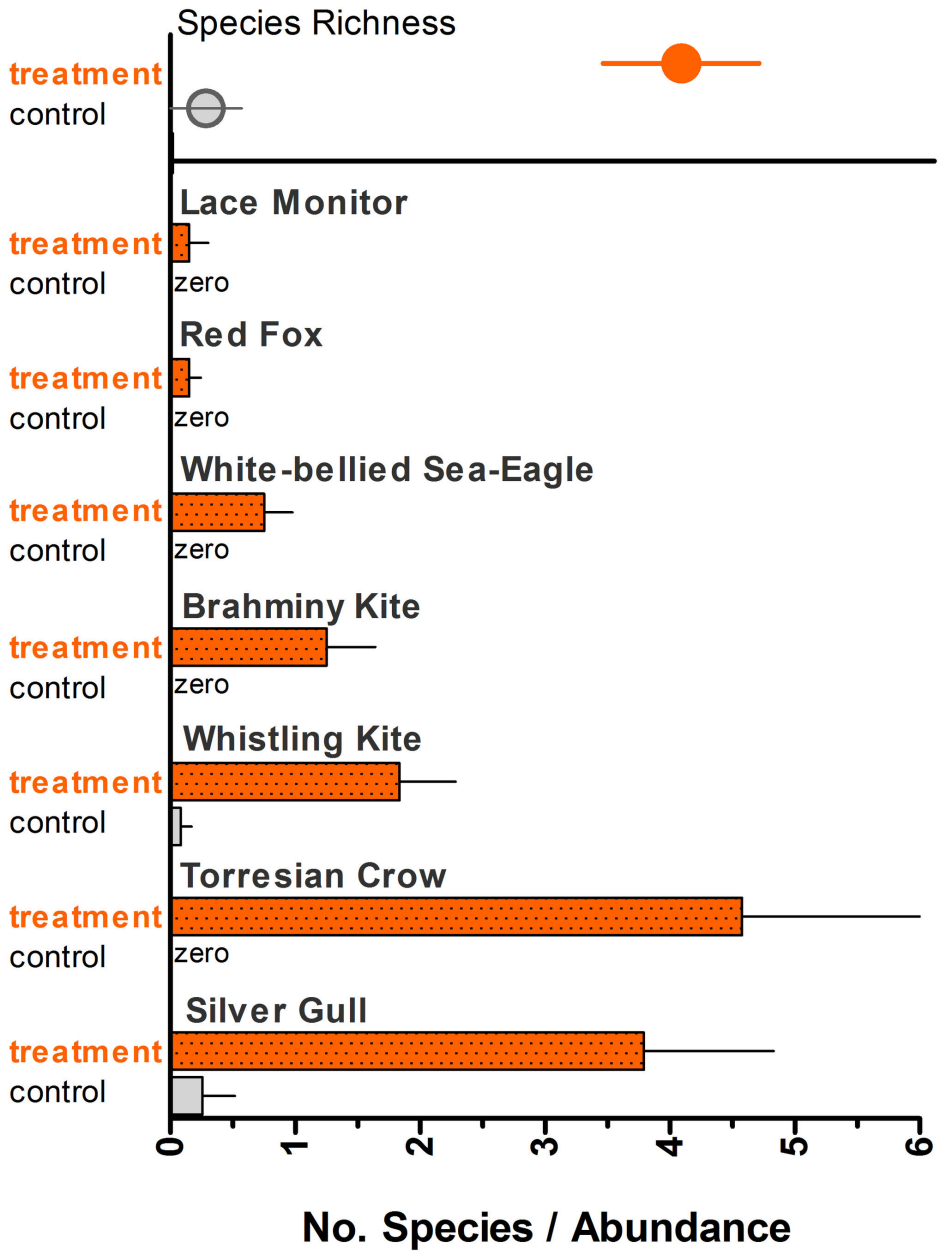
Comparison of species richness (top panel) and abundance of vertebrate scavengers between experimental plots to which fish carcasses were added (treatment) and in unaltered plots (‘control’). The measure of abundance is the mean number of individuals recorded per 24 h of camera deployment.

**Figure 5:**
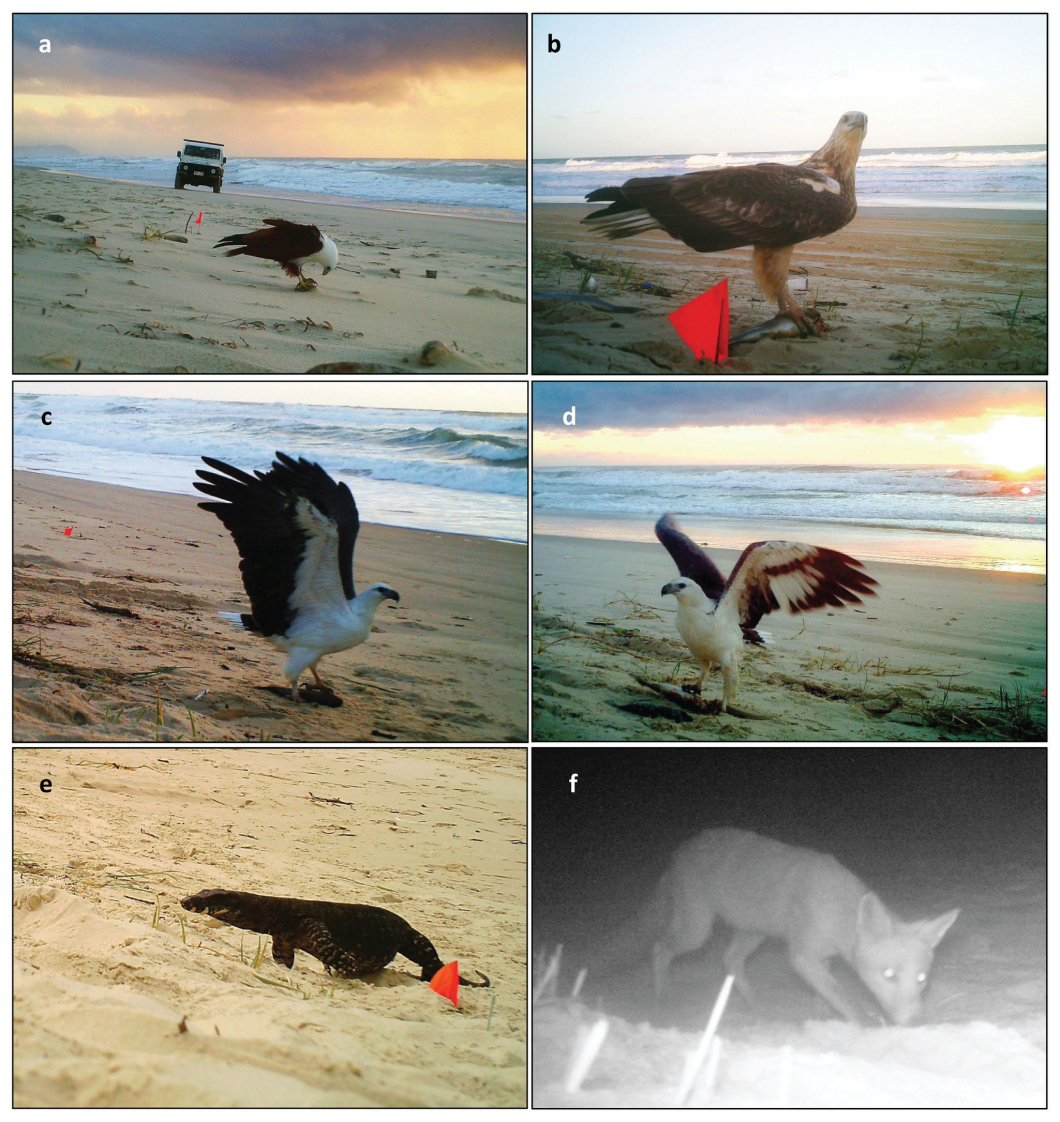
Vertebrate scavengers on the beach. a) Brahminy kite consuming a mullet; b) Whistling kite gripping a fish; c & d) White-bellied sea eagle starting to lift a fish carcass off the beach; e) Lace monitor approaching carrion; f) Red fox at an experimental plot containing fish carcasses.

**Figure 6:**
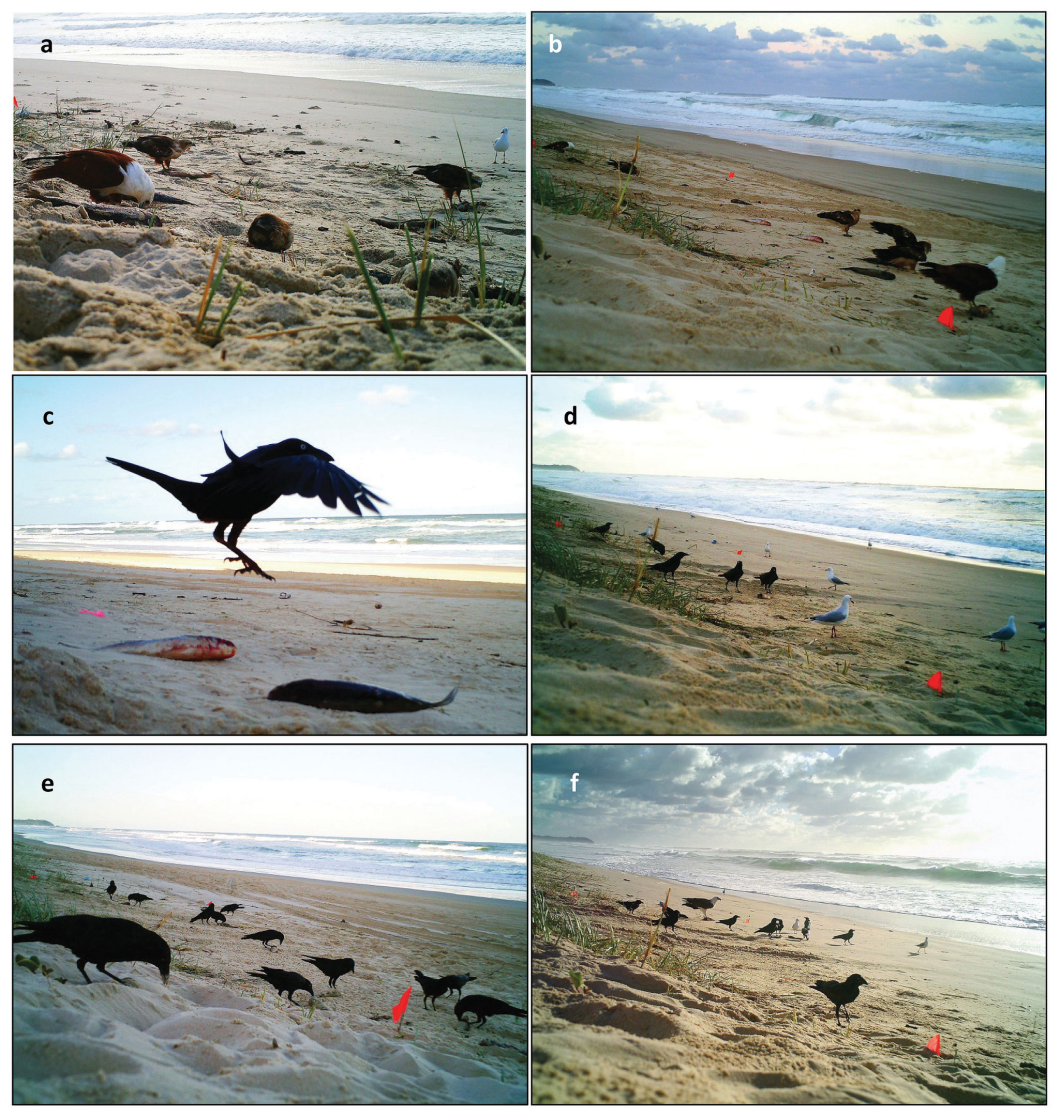
Aggregative responses of avian scavengers to fish carcasses at the beach-dune interface. a & b) Mixed flock of two brahminy kites and three whistling kites feeding on fish carrion in an area of approximately 3 x 10 m; b) Torresian crow reacting to freshly-deposited fish carrion; c & d) flocks of crows and silver gulls amassing around fish carrion; f) mixed flock of avian scavengers, with a white-bellied sea eagle amongst crows and gulls.

Vertebrate scavengers, dominated numerically and functionally by birds, reacted quickly and strongly to enhanced carrion availability (Figures 4-6, Table 4). Torresian crows and silver gulls were the most abundant avian scavengers and they accounted for half of all scavenging records captured with the automated cameras (Table 4). Each of the three species of raptors (whistling kite, brahminy kite, and white-bellied sea eagle) were common, occurring at all but one plot to which carcasses had been added (Figures 5 & 6). These raptors foraged as facultative scavengers and comprised a substantial proportion (43%) of scavenging bouts recorded (Table 4). There were three records of red foxes and one of a lace monitor scavenging in the plots (Table 4, Figure 5).

**Table 4:** Metrics of vertebrate scavenger occurrence, abundance and feeding activity in experimental plots that received fish carcasses (‘carrion’) or were left unaltered (‘control’).

**Species**	**Treatment**	**Sightings^1^**	**Feeding Records^2^**		**Max. No. individuals per camera deployment^3^**	**Time spent at carcasses^4^**	
			**n**	**(%)**		**mean**	**max**
Torresian Crow							
	control	0	1	0%	1	0	0
	treatment	8	75	28%	13	46	281
Silver Gull							
	control	1	2	1%	2	1	5
	treatment	9	62	23%	9	22	64
Whistling Kite							
	control	1	1	0%	1	0	0
	treatment	8	53	20%	3	14	44
Brahminy Kite							
	control	0	0	0%	0	0	0
	treatment	8	23	9%	2	8	24
White-bellied Sea Eagle							
	control	0	0	0%	0	0	0
	treatment	8	38	14%	1	4	14
Red Fox							
	control	0	0	0%	0	0	0
	treatment	3	5	2%	1	1	5
Lace Monitor							
	control	0	0	0%	0	0	0
	treatment	1	5	2%	1	1	6

1 The number of camera deployments in which a species was sighted (n);

2 The number, and proportion, of records where scavengers fed on carrion (n, %);

3 Largest number of individuals seen per deployment site (n);

4 Sum of all recorded feeding bouts per 24h of camera deployment (minutes).

The avian scavengers displayed a remarkable aggregative response to carrion patches (Figures 4-6). We regularly observed both raptors and non-raptors scavenging on carcasses at the dune-beach interface shortly after fish had been deposited there (Figure 6). This response was spatially distinctive, concentrated around carrion resources, creating nuclei of often intense bird scavenging along the strandline of the beach (Figure 6). We observed only three birds in control plots without carrion (Table 4, Figure 4), demonstrating that the large numbers seen in carrion patches were not an experimental artefact (i.e., consistent attraction of many individuals to cameras or to footprints).

**Figure 7:**
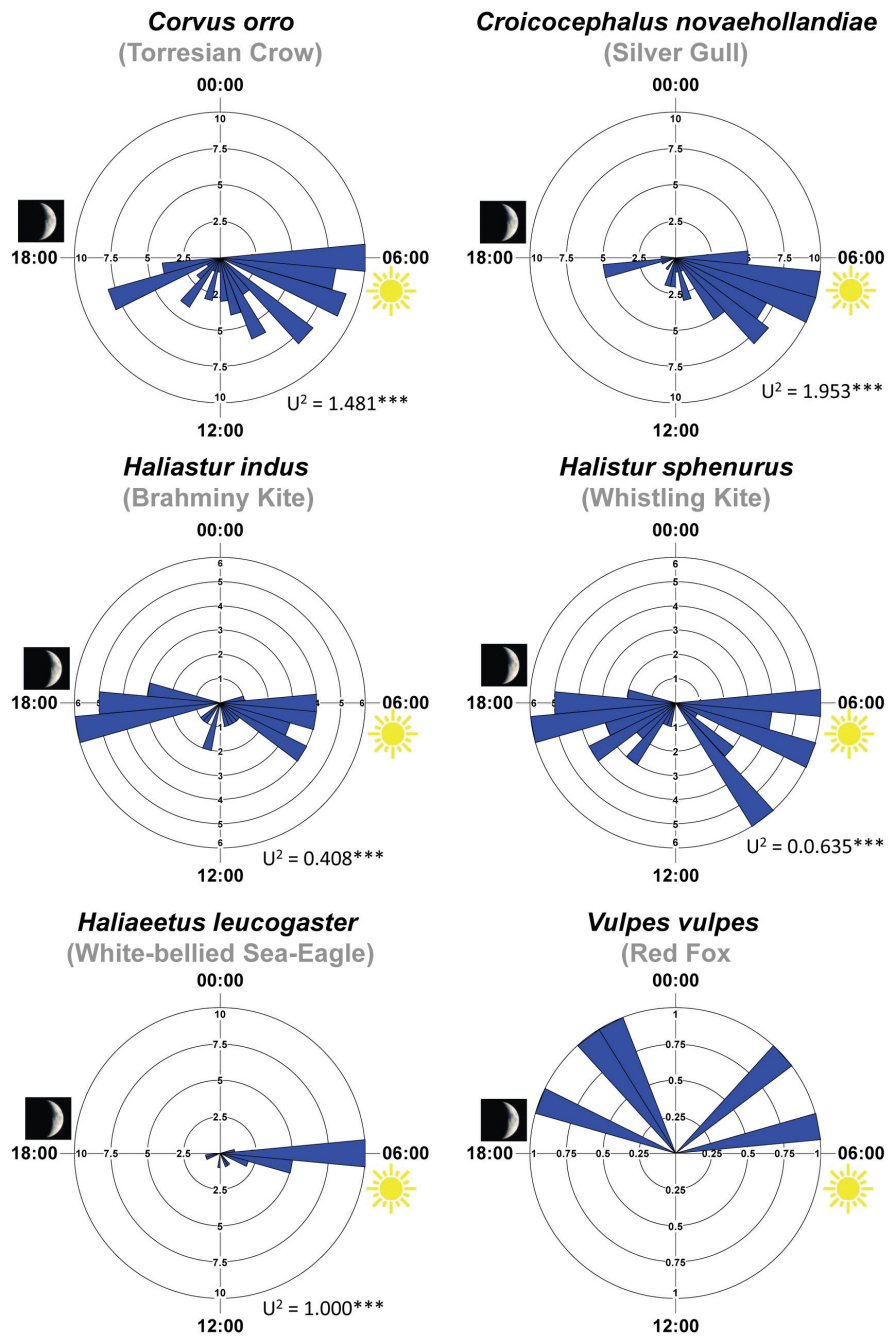
Temporal distribution of scavenging activity in experimental plots by the six vertebrate consumers recorded. Sunrise was close to 06:00 and sunset close to 18: 00 during the experiment (U2 is the test statistics for Watson’s test of circular uniformity; *** p < 0.001).

Feeding by avian scavengers was generally most intense at sunrise (especially in white-bellied sea eagles) and a few hours afterwards in the morning (Figure 7). Both species of kites exhibited a second peak of scavenging activity before and at dusk; foxes scavenged only at night (Figure 7). The avian scavengers also differed in their feeding behaviour at carcasses. White-bellied sea eagles, the largest raptor, can take whole fish carcasses from the beach in a ‘swooping’ motion, spending little or no time on the ground. By contrast, the two smaller kite species were often seen feeding on fish whilst on the ground, generally opening the body cavity of fish first to access the entrails. Crows and gulls seemed unable to lift entire carcasses and hence fed for longer times in situ, often in sizeable flocks (Table 5, Figure 6). Crows were also aggressive against all three raptor species, aiming to defend resource-rich patches which they tried to monopolise. All avian scavengers detected carrion at approximately the same frequency and speed (Table 5).

**Table 5:** Comparison of avian scavenger species in terms of their capacity to detect carcasses (being the first species to arrive at carcass and time to detection) and the time spent per feeding bout in experimental plots containing carrion.

	**No. times first at carcass**	**Detection time (h, mean)**		**Duration of feeding bout (sec)**			**Tukey’s HSD**
				**n**	**mean**	**se**	
White-bellied Sea Eagle	4	1.7		23	322	298	a
Brahminy Kite	5	5.0		38	372	232	a
Whistling Kite	7	1.4		53	509	196	a,b
Silver Gull	4	2.5		62	680	182	a,b
Torresian Crow	5	2.7		75	1219	165	b

Tukey’s HSD refers to post-hoc testing following a significant main effect for species in Anova comparing the mean duration of feeding bouts among species (F_4,250_ = 3.84, p 0.005).

## Discussion

Enhanced availability of fish carcasses at the beach-dune interface resulted in a number of trophic responses, characterised most saliently by four, interrelated processes: 1) increased numbers of scavengers in distinct aggregations at carcasses, 2) fast and near-complete removal of available fish carrion by scavengers, 3) intense consumption by a vertebrate scavenger guild that was numerically and functionally dominated by birds, and 4) fluxes of marine matter across the beach-dune interface with terrestrial scavengers as vectors.

### Aggregative responses of scavengers to carcasses

Carrion forms part of the material imported from the ocean that underpins food webs on sandy beaches [12]. Two distinct scavenger responses to concentrated and pulsed food falls can occur: populations increase in size (true numerical response) or individuals concentrate temporarily around carrion (aggregative response). The time span of our experiment permitted demonstration of aggregative responses, which we detected in both invertebrate (ghost crabs) and vertebrate (birds, mammals, reptiles) consumers of carcasses.

Ghost crabs (*Ocypode* spp.) have a catholic diet, locating carrion using chemoreceptors to detect low-molecular-weight compounds (e.g. hydrogen sulphide and putrescine) that emanate from rotting animal flesh [53,54]. They can also be biological vectors in situations where they feed on marine matter on the unvegetated part of the beach and transfer part of this material landwards when retreating to burrows located near or in the dunes [31]. We measured, for the first time, a significant and substantial aggregative response in the ghost crab population to carrion augmentation (Figure 2), demonstrating experimentally that beach invertebrates are well-adapted (sensu [55]) to capitalize on pulsed and spatially-concentrated inputs of food from the ocean.

The aggregative response by the invertebrate scavengers was most pronounced in the largest crabs, which moved to experimental food patches in comparatively larger numbers than smaller crabs. Ghost crabs are prey for raptors [56], crows and black-headed terns (Schlacher pers. obs), foxes [57], and monitor lizards [58] when these forage on beaches. We recorded all of these vertebrates and observed some (crows, terns) hunting for crabs on the beach surface. The observed size-dependent response to carrion may be explained by a lower risk for larger individuals to be predated by birds. Alternatively, larger individuals may be more capable of movingto carcasses and could also prevent smaller crabs from burrowing near carcasses; further experimentation is needed to distinguish between these possibilities.

True reproductive responses to trophic subsidies in coastal areas have been recorded for both invertebrate and vertebrate consumers. For example, Spiller [59] demonstrated that orb spiders (*Zygiella x-notata*) were more fecund when more prey was available on the seashore. Coastal populations of arctic foxes ( *Vulpes* *lagopus* ) also respond positively to marine subsidies [60]. In our situation it is plausible that carrion addition experiments over long periods could elicit a numerical response in the scavenger populations. Since these effects are primarily contingent on the generation time of the consumers, they would be in the order of several months for ghost crabs and longer again for birds of prey. For example, whistling kites and brahminy kites mature at 1-2 years, and white-bellied sea eagles can take 3-7 years to reach adulthood [61]. Nevertheless it is not improbable that sustained higher inputs of carrion to the beach would elicit enhanced reproductive output in avian and mammalian scavengers, complementing the strong aggregative response seen here.

### Rapid and efficient removal of carrion

Many ecosystems contain unexpectedly large numbers of dead animals that have died from non-predation events, such as disease, malnutrition, exposure to extreme weather events, mass parasites, and accidents [62]. Because beaches are depositional environments, clearly illustrated by the accumulations of wrack [16], we expect that the deposition of carrion on the strandline is not an infrequent event. Smaller carrion on beaches is, however, not widely reported in the literature. This could be due to low standing stocks of carrion despite high input rates when detection and consumption of freshly-deposited carrion items is very rapid and efficient [63]. Alternatively, comparatively smaller carrion, such as the mullet used in our study, is neglected because strandings of larger carcasses (i.e. cetacean and pinnipeds) are perceived as the main form of carrion supply to beaches [64].

Carcass use (97% removed over 8 days) on sandy beaches rivals that of other ecosystems. Scavenging rates have been measured in several systems, generally reporting high proportions of carcasses removed. For example, 97% of brown chicken (*Gallus gallusdomesticus*) carcasses placed in a tropical forest were consumed within 3 days [65]. In grasslands of South Dakota, USA, striped skunks ( *Mephitis* *mephitis* ) fed on small bird carcasses at 66% removal efficiency [66]. In the evergreen shrublands of Chile, didelphids ( *Marmosa* *elegans* ) scavenged 100% of the rodent carcasses that were experimentally added [67]. Raccoons ( *Procyon* *lotor* ), grey foxes ( *Urocyon* *cinereoargenteus* ) and feral pigs (*Sus scrofa*) scavenged 65% of house mouse carrion in North American hardwood forests [68]. In the Arctic Tundra, 99% of lemmings ( *Dicrostox* *richardsoni* ) were scavenged by red foxes ( *Vulpes* *vulpes* ) and arctic foxes ( *Vulpes* *lagopus* ) in one season [64].

Scavengers have evolved to detect and consume carrion efficiently [7]. We found that terrestrial avian scavengers had consumed nearly all of the fish carrion deposited on the beach within a week. Two factors appear most likely to contribute to this rapid and efficient scavenging on sandy beaches: a) raptors and other avian scavengers are abundant along the shoreline [69,70], and appear to have a specific search behaviour where individuals fly parallel to the base of the dunes where most carrion naturally strands (Schlacher, pers. obs.), and b) dead animals stranded on beaches are generally highly visible (ready detection) and scavengers have unimpeded access to them.

### Birds dominate the vertebrate scavenger guild

The majority of the feeding records by vertebrate scavengers throughout our study were from birds. Flight enables birds to search large areas and detect patchy resources (i.e. carcass falls) in ways generally not possible for other species [71]. Soaring is especially energy-efficient, conferring an advantage to large, scavenging birds [72]. We found large raptors to be important consumers of carrion at the strandline of beaches: they comprised half of all recorded scavenging events captured with the automated cameras during the experiment and they frequently carried whole fish off the beach into the dunes.

Birds other than raptors and vultures can, however, also be important scavengers on a diversity of carcass types [41,73]. Torresian crows were frequently observed at the fish carrion and are known to forage on coastal dunes and beaches [29,74], where they prey on insects, invertebrates, small vertebrates, eggs, carrion, and food scraps [75,76]. We observed mobbing of raptors and other aggressive behaviours where crows actively defended carcass patches against raptors and monopolised carrion resources at times (Figure 6). Their feeding behaviour also differs from raptors: the smaller species (crows and gulls) remain feeding on the beach, pecking at carcasses for long periods, whereas larger raptors (e.g. white-bellied sea eagles) spend comparatively less time on the shore and can carry whole fish into the dunes.

Marine carrion is an important dietary component in many carnivorous terrestrial mammals that feed on the seashore [77], subsidising local populations as demonstrated by the seminal work of Rose and Polis [57] on coyotes. In our situation, we observed red foxes ( *Vulpes* *vulpes* ) scavenging along the shoreline at night. We also recorded fox prints in treatment plots and tracks that ran parallel to the shore, in what may have been part of the fox’s territory, where the animal displays a nocturnal traversing behaviour [78]. Significantly, there is temporal resource partitioning between foxes and avian scavengers on the beach, with foxes scavenging nocturnally and birds diurnally.

Our record of a large carnivorous lizard (lace monitor, *Varanus* *varius* ) being attracted to fish carrion is interesting because there are few published records of terrestrial reptiles scavenging on beaches (but see 79,80). Yet lace monitors are known to occur in coastal dunes and on beaches, especially near camp grounds where they feed on food scraps (Schlacher pers. obs. [81]). Being carnivores that incorporate a broad prey spectrum (e.g. birds, fish, mammals, amphibians, eggs, and insects) dominated by carrion [82], the trophic role of these large lizards, and perhaps reptiles more generally, may be underappreciated in dune and beach food webs.

### Possible ecosystem consequence of scavenging

Cross-boundary exchanges are ubiquitous, representing donor-controlled subsidies of energy and nutrients that can modify the architecture and dynamics of recipient systems [1,6]. These fluxes are important in sandy beaches, systems that have little *in situ* productivity and receive marine input across long and open interfaces with the ocean [83]. In a spatial context, movements of material across ecological interfaces connect ecosystems across a range of scales [2,84].

Such spatial coupling is probable in the system studied here, chiefly because of the mobility of consumers across the interface region of interest. Generally, mobile organisms that cross boundaries and transfer material between systems can be important biological vectors [85,86]. All the scavengers examined in this experiment are mobile and switch between abutting habitats. Ghost crabs feed on marine matter [31], their foraging distribution often concentrated in the swash zone low on the beach or along the strandline at the beach-dune interface (Schlacher pers. obs.). They can act as biological vectors in spatial ecosystem coupling when transferring material foraged on the beach to burrows located in the dunes [87].

Birds are important vectors in the transport of marine nutrients to terrestrial systems [86]. In our experiment, the bulk of marine carrion removal from the beach was done by avian scavengers of primarily terrestrial provenance. All bird species that were found to consume marine carrion at the land-ocean ecotone roost and breed in the dunes [61], thereby transporting nutrients (e.g. faeces) and organic matter (e.g. carcasses and other prey) upland from the beach. White-bellied sea eagles illustrate the functional role of birds as biological vector in these habitats particularly well: they habitually target and lift fish from the surf-zone of beaches and carry them inland into the dunes to be eaten: this is a prime example of organic matter transfer by a biological vector across an ecotone. Because of messy feeding and the passing of faecal matter [36], effects of this ecotonal coupling could propagate beyond the individual predator.

## Conclusions

Our experiment showed unequivocally that increased carrion availability at the beach-dune interface results in quick and substantial aggregative responses of scavengers to carcasses. These trophic subsidy effects, resulting from imports of marine animal carcasses, are measurable at multiple levels of the food web. Terrestrial raptors, lace monitors, and foxes efficiently consumed virtually all marine necromass within a few days, demonstrating the potential for substantial ecotonal coupling in this system. Birds dominated the scavenging activity of the vertebrates, with a minor contribution by foxes and reptilian scavengers. High mobility of terrestrial raptors that concentrate along the shoreline and their efficient scavenging habits on marine carrion confer a capacity to transfer marine matter across the beach-dune interface, forming a biological vector linking ocean productivity with terrestrial food webs. An important aspect of these predicted energetic linkages across habitat boundaries is the spatial ambit of trophic cross-boundary effects. Thus, future work may involve tracking raptor species to determine the spatial extent of marine subsidies at the land-ocean interface.
